# Trichodermol (4α-hydr­oxy-12,13-epoxy­trichothec-9-ene)

**DOI:** 10.1107/S1600536809044080

**Published:** 2009-10-28

**Authors:** Jing-Li Cheng, Yong Zhou, Fu-Cheng Lin, Jin-Hao Zhao, Guo-Nian Zhu

**Affiliations:** a College of Agriculture and Biotechnology, Zhejiang University, Hangzhou 310029, People’s Republic of China; b Institute of Biotechnology, Zhejiang University, Hangzhou 310029, People’s Republic of China

## Abstract

In the title compound, C_15_H_22_O_3_, the five-membered ring displays an envelope conformation, whereas the two six-membered rings show different conformations, *viz*. chair and half-chair. In the crystal, mol­ecules are linked through inter­molecular O—H⋯O hydrogen bonds, forming chains running along the *b* axis.

## Related literature

For the fungicidal activity of the endophytic fungus *Trichoderma taxi sp. nov.* from *Taxus mairei*, see: Nielsen *et al.* (2005 ▶); Zhang *et al.* (2007 ▶). For the related Trichodermin structure, see: Chen *et al.* (2008 ▶). For the extinction correction, see: Larson (1970 ▶).
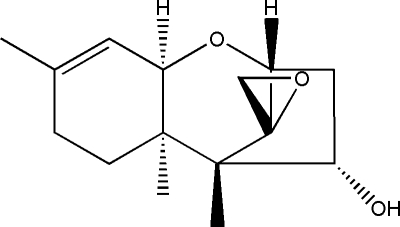


## Experimental

### 

#### Crystal data

C_15_H_22_O_3_
*M* *_r_* = 250.34
Monoclinic, 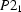

*a* = 6.8284 (2) Å
*b* = 6.6209 (3) Å
*c* = 14.7170 (6) Å
β = 96.7507 (11)°
*V* = 660.74 (4) Å^3^
*Z* = 2
Mo *K*α radiation
μ = 0.09 mm^−1^
*T* = 296 K
0.66 × 0.49 × 0.28 mm

#### Data collection

Rigaku R-AXIS RAPID IP diffractometer
Absorption correction: multi-scan (*ABSCOR*; Higashi, 1995 ▶) *T*_min_ = 0.934, *T*_max_ = 0.976
6503 measured reflections
1634 independent reflections
1540 reflections with *F* ^2^ > 2σ(*F* ^2^)
*R*_int_ = 0.018

#### Refinement

*R*[*F* ^2^ > 2σ(*F* ^2^)] = 0.034
*wR*(*F* ^2^) = 0.095
*S* = 1.00
1634 reflections
164 parameters
H-atom parameters constrained
Δρ_max_ = 0.17 e Å^−3^
Δρ_min_ = −0.15 e Å^−3^

### 

Data collection: *PROCESS-AUTO* (Rigaku, 1998 ▶); cell refinement: *PROCESS-AUTO*; data reduction: *CrystalStructure* (Rigaku/MSC, 2002 ▶); program(s) used to solve structure: *SIR97* (Altomare *et al.*, 1993 ▶); program(s) used to refine structure: *CRYSTALS* (Watkin *et al.*, 1996 ▶); molecular graphics: *ORTEP-3 for Windows* (Farrugia, 1997 ▶); software used to prepare material for publication: *WinGX* (Farrugia, 1999 ▶) and *PLATON* (Spek, 2009 ▶).

## Supplementary Material

Crystal structure: contains datablocks global, I. DOI: 10.1107/S1600536809044080/si2217sup1.cif

Structure factors: contains datablocks I. DOI: 10.1107/S1600536809044080/si2217Isup2.hkl

Additional supplementary materials:  crystallographic information; 3D view; checkCIF report

## Figures and Tables

**Table 1:** Hydrogen-bond geometry (Å, °)

*D*—H⋯*A*	*D*—H	H⋯*A*	*D*⋯*A*	*D*—H⋯*A*
O2—H201⋯O1^i^	0.84	2.02	2.839 (2)	165

Symmetry code: (i) 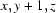.

**Fig. 1.**
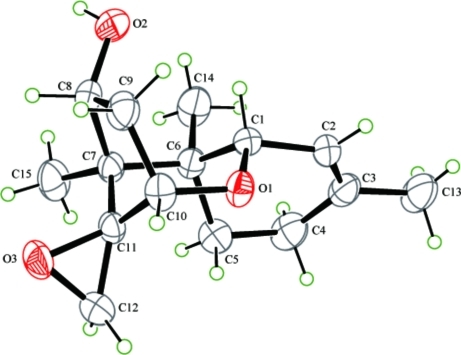
The molecular structure of (I), showing the atom-labelling scheme. Displacement ellipsoids are drawn at the 30% probability level.

**Fig. 2.**
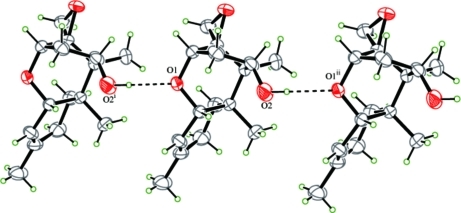
View showing the O—H···O hydrogen bonding (dashed lines). Symmetry code: (i) = (1+x, y, 1+z).

## supplementary crystallographic information

### Comment

The endophytic fungi *Trichoderma taxi sp. nov.* from *Taxus mairei* can produce a compound with fungicidal activity-Trichodermin (Zhang *et al.*, 2007), which is a member of the 4β-aceoxy-12,13-epoxytrichothecene family (Nielsen *et al.*, 2005). Bioassays showed Trichodermin strongly inhibited Rhizoctonia solani and Botrytis cinere. In order to find the relationship between the stereochemistry of the C4 position and biological activities, the title compound had been designed and synthesized. Its molecular structure is shown in Fig. 1. In the molecule, the five membered ring displays an envelope conformation with atom C11 at the flap position 0.715 (3) Å out of the mean plane formed by the other four atoms. The O1-containing six-membered ring displays a chair conformation. The typical C2=C3 double bond length of 1.325 (2) Å suggests that C2 and C3 atoms are sp^2^ hybridized, which correlates with the larger C1—C2—C3 bond angle of 124.36 (16) ° and C2—C3—C4 bond angle of 122.95 (18) ° and a small C1—C2—C3—C4 torsion angle of -3.0 (3) °, as compared to 3.0 (3) ° in the compound of Trichodermin. And the C3-containing six-membered ring displays a half-chair conformation, as well as the compound of Trichodermin (Chen *et al.*). There are intermolecular O—H···O hydrogen bonds (Table 1) in the crystal structure, which lead to the formation of chains running along the *b* axis (Fig. 2).

### Experimental

To a solution of 12,13-Epoxytrichothec-9-ene-4-one (1 g) in THF(100 ml) containing 10 ml of methanol was added sodium borohydride (100 mg) and the reactant was partitioned between 100 ml of ethyl acetate and water. The organic layer was dried with MgSO_4_ and concentrated, and the residue was chromatographed to give 620 mg solid precipitate. The solid was filtrated and recrystallized with 95% ethanol to colourless blocks.

[α]D = 65.7 (c 0.052). ESI-MS: 251 (M+H)+ (100%); 1H-NMR (500 MHz, CDCl3, ppm): 5.46 (1H, d, J=5.5Hz, H-10), 4.27 (1H, t, H-4), 4.22 (1H, d, J=5.5Hz, H-2), 3.67 (1H, d, J=5.5Hz, H-11), 3.05 (1H, d, J=4.0Hz, H-13), 2.78 (1H, d, J=4.0Hz, H-13), 2.56-2.49 (1H, m, H-3), 2.00-1.97 (2H, m, H-8), 1.97-1.96 (1H, m, H-3), 1.96-1.94 (1H, m, H-7), 1.71 (3H, s, H-16), 1.33-1.31 (1H, m, H-7), 1.09 (3H, s, H-14), 0.86 (3H, s, H-15).

### Refinement

In the absence of significant anomalous scattering effects, Friedel pairs were averaged; the absolute configuration was not determined. The H atoms were geometrically placed (C—H = 0.93–0.98 Å) and refined as riding with *U*_iso_(H) = 1.2*U*_eq_(C) or 1.5*U*_eq_(methyl C). The methyl group was allowed to rotate, but not to tip, to best fit the electron density.

### Crystal data

**Table:**

C_15_H_22_O_3_	*F*(000) = 272.00
*M**_r_* = 250.34	*D*_x_ = 1.258 Mg m^−^^3^
Monoclinic, *P*2_1_	Mo *K*α radiation, λ = 0.71075 Å
Hall symbol: P 2yb	Cell parameters from 6124 reflections
*a* = 6.8284 (2) Å	θ = 3.0–27.4°
*b* = 6.6209 (3) Å	µ = 0.09 mm^−^^1^
*c* = 14.7170 (6) Å	*T* = 296 K
β = 96.7507 (11)°	Chunk, colorless
*V* = 660.74 (4) Å^3^	0.66 × 0.49 × 0.28 mm
*Z* = 2	

### Data collection

**Table:**

Rigaku R-AXIS RAPID IP diffractometer	1540 reflections with *F*^2^ > 2σ(*F*^2^)
Detector resolution: 10.00 pixels mm^-1^	*R*_int_ = 0.018
ω scans	θ_max_ = 27.4°
Absorption correction: multi-scan (*ABSCOR*; Higashi, 1995)	*h* = −7→8
*T*_min_ = 0.934, *T*_max_ = 0.976	*k* = −8→8
6503 measured reflections	*l* = −19→18
1634 independent reflections	

### Refinement

**Table:**

Refinement on *F*^2^	*w* = 1/[0.0012*F*_o_^2^ + 1.5σ(*F*_o_^2^)]/(4*F*_o_^2^)
*R*[*F*^2^ > 2σ(*F*^2^)] = 0.034	(Δ/σ)_max_ < 0.001
*wR*(*F*^2^) = 0.095	Δρ_max_ = 0.17 e Å^−^^3^
*S* = 1.00	Δρ_min_ = −0.15 e Å^−^^3^
1634 reflections	Extinction correction: Larson (1970), equation 22
164 parameters	Extinction coefficient: 184 (28)
H-atom parameters constrained	

### Special details

**Table:**

Geometry. ENTER SPECIAL DETAILS OF THE MOLECULAR GEOMETRY
Refinement. Refinement using all reflections. The weighted *R*-factor (*wR*) and goodness of fit (*S*) are based on *F*^2^. *R*-factor (gt) are based on *F*. The threshold expression of *F*^2^ > 2.0 σ(*F*^2^) is used only for calculating *R*-factor (gt).

### Fractional atomic coordinates and isotropic or equivalent isotropic displacement parameters (Å^2^)

**Table:**

	*x*	*y*	*z*	*U*_iso_*/*U*_eq_	
O1	0.84609 (16)	0.3577 (2)	0.83213 (8)	0.0329 (3)	
O2	0.9112 (2)	0.9341 (2)	0.83678 (11)	0.0535 (4)	
O3	0.44991 (17)	0.5728 (2)	0.95141 (8)	0.0428 (3)	
C1	0.8680 (2)	0.4910 (2)	0.75578 (11)	0.0295 (4)	
C2	0.9296 (2)	0.3595 (3)	0.68075 (12)	0.0389 (4)	
C3	0.8120 (2)	0.3061 (3)	0.60656 (12)	0.0423 (5)	
C4	0.5978 (2)	0.3670 (3)	0.59348 (12)	0.0484 (5)	
C5	0.5275 (2)	0.4606 (3)	0.67912 (12)	0.0394 (4)	
C6	0.6784 (2)	0.6115 (2)	0.72646 (11)	0.0307 (4)	
C7	0.6045 (2)	0.7082 (2)	0.81427 (11)	0.0298 (4)	
C8	0.7735 (2)	0.8181 (2)	0.87935 (12)	0.0374 (4)	
C9	0.8824 (2)	0.6492 (3)	0.93698 (12)	0.0398 (4)	
C10	0.7723 (2)	0.4555 (3)	0.90883 (11)	0.0326 (4)	
C11	0.5685 (2)	0.5348 (2)	0.87747 (11)	0.0294 (4)	
C12	0.3896 (2)	0.4194 (3)	0.88380 (12)	0.0418 (5)	
C13	0.8819 (4)	0.1859 (4)	0.52998 (14)	0.0596 (6)	
C14	0.7229 (3)	0.7762 (3)	0.65783 (14)	0.0505 (5)	
C15	0.4273 (2)	0.8481 (3)	0.79217 (14)	0.0485 (5)	
H1	0.9748	0.5866	0.7745	0.035*	
H2	1.0589	0.3125	0.6868	0.047*	
H8	0.7109	0.9068	0.9207	0.045*	
H10	0.7711	0.3629	0.9607	0.039*	
H41	0.5787	0.4649	0.5442	0.058*	
H42	0.5188	0.2479	0.5769	0.058*	
H51	0.4041	0.5308	0.6617	0.047*	
H52	0.5066	0.3534	0.7219	0.047*	
H91	1.0183	0.6401	0.9241	0.048*	
H92	0.8794	0.6749	1.0017	0.048*	
H121	0.3980	0.2778	0.9006	0.050*	
H122	0.2745	0.4411	0.8396	0.050*	
H131	0.8038	0.0656	0.5201	0.072*	
H132	1.0178	0.1497	0.5458	0.072*	
H133	0.8688	0.2656	0.4751	0.072*	
H141	0.7607	0.7139	0.6036	0.061*	
H142	0.8285	0.8602	0.6850	0.061*	
H143	0.6072	0.8572	0.6421	0.061*	
H151	0.3317	0.7839	0.7485	0.058*	
H152	0.4697	0.9723	0.7670	0.058*	
H153	0.3691	0.8760	0.8471	0.058*	
H201	0.8723	1.0539	0.8294	0.066*	

### Atomic displacement parameters (Å^2^)

**Table:**

	*U*^11^	*U*^22^	*U*^33^	*U*^12^	*U*^13^	*U*^23^
O1	0.0392 (5)	0.0274 (6)	0.0324 (5)	0.0083 (5)	0.0063 (4)	−0.0003 (5)
O2	0.0587 (8)	0.0287 (7)	0.0781 (10)	−0.0102 (7)	0.0296 (7)	−0.0093 (7)
O3	0.0493 (7)	0.0455 (8)	0.0377 (6)	0.0024 (6)	0.0226 (5)	−0.0016 (6)
C1	0.0308 (7)	0.0267 (9)	0.0320 (7)	−0.0024 (6)	0.0076 (5)	−0.0016 (6)
C2	0.0398 (8)	0.0335 (10)	0.0466 (9)	−0.0012 (8)	0.0179 (7)	−0.0075 (8)
C3	0.0633 (10)	0.0326 (10)	0.0344 (9)	−0.0079 (9)	0.0207 (8)	−0.0044 (8)
C4	0.0632 (11)	0.0501 (13)	0.0308 (8)	−0.0053 (11)	0.0004 (7)	−0.0065 (9)
C5	0.0381 (7)	0.0463 (12)	0.0330 (8)	0.0009 (8)	0.0007 (6)	0.0002 (9)
C6	0.0386 (7)	0.0272 (9)	0.0273 (7)	0.0021 (7)	0.0083 (5)	0.0025 (6)
C7	0.0336 (7)	0.0241 (8)	0.0329 (8)	0.0043 (6)	0.0093 (5)	0.0022 (7)
C8	0.0424 (8)	0.0303 (10)	0.0419 (9)	−0.0012 (7)	0.0153 (7)	−0.0106 (8)
C9	0.0393 (8)	0.0459 (11)	0.0337 (8)	0.0021 (8)	0.0023 (6)	−0.0101 (8)
C10	0.0383 (7)	0.0342 (10)	0.0254 (7)	0.0076 (7)	0.0049 (5)	0.0043 (7)
C11	0.0343 (7)	0.0293 (9)	0.0261 (7)	0.0030 (7)	0.0103 (5)	0.0001 (7)
C12	0.0393 (8)	0.0427 (11)	0.0455 (9)	−0.0034 (8)	0.0145 (7)	0.0013 (9)
C13	0.0909 (15)	0.0460 (13)	0.0478 (11)	−0.0127 (13)	0.0322 (11)	−0.0138 (11)
C14	0.0785 (13)	0.0354 (11)	0.0413 (10)	0.0041 (10)	0.0229 (9)	0.0086 (9)
C15	0.0516 (9)	0.0397 (12)	0.0563 (11)	0.0188 (10)	0.0144 (8)	0.0089 (10)

### Geometric parameters (Å, °)

**Table:**

O1—C1	1.450 (2)	C1—H1	0.980
O1—C10	1.443 (2)	C2—H2	0.930
O2—C8	1.416 (2)	C4—H41	0.970
O3—C11	1.453 (2)	C4—H42	0.970
O3—C12	1.447 (2)	C5—H51	0.970
C1—C2	1.504 (2)	C5—H52	0.970
C1—C6	1.539 (2)	C8—H8	0.980
C2—C3	1.325 (2)	C9—H91	0.970
C3—C4	1.508 (2)	C9—H92	0.970
C3—C13	1.503 (3)	C10—H10	0.980
C4—C5	1.531 (2)	C12—H121	0.970
C5—C6	1.542 (2)	C12—H122	0.970
C6—C7	1.577 (2)	C13—H131	0.960
C6—C14	1.541 (2)	C13—H132	0.960
C7—C8	1.587 (2)	C13—H133	0.960
C7—C11	1.516 (2)	C14—H141	0.960
C7—C15	1.528 (2)	C14—H142	0.960
C8—C9	1.541 (2)	C14—H143	0.960
C9—C10	1.520 (2)	C15—H151	0.960
C10—C11	1.508 (2)	C15—H152	0.960
C11—C12	1.453 (2)	C15—H153	0.960
O2—H201	0.840		
			
C1—O1—C10	114.24 (13)	C3—C4—H42	108.5
C11—O3—C12	60.14 (11)	C5—C4—H41	108.5
O1—C1—C2	106.26 (14)	C5—C4—H42	108.5
O1—C1—C6	111.87 (12)	H41—C4—H42	109.5
C2—C1—C6	113.14 (12)	C4—C5—H51	108.8
C1—C2—C3	124.36 (16)	C4—C5—H52	108.8
C2—C3—C4	121.26 (18)	C6—C5—H51	108.8
C2—C3—C13	122.95 (18)	C6—C5—H52	108.8
C4—C3—C13	115.78 (17)	H51—C5—H52	109.5
C3—C4—C5	113.36 (15)	O2—C8—H8	108.1
C4—C5—C6	112.11 (15)	C7—C8—H8	108.1
C1—C6—C5	106.63 (14)	C9—C8—H8	108.1
C1—C6—C7	108.69 (12)	C8—C9—H91	110.4
C1—C6—C14	109.07 (15)	C8—C9—H92	110.4
C5—C6—C7	111.85 (13)	C10—C9—H91	110.4
C5—C6—C14	109.60 (14)	C10—C9—H92	110.4
C7—C6—C14	110.88 (15)	H91—C9—H92	109.5
C6—C7—C8	113.63 (13)	O1—C10—H10	111.4
C6—C7—C11	106.61 (14)	C9—C10—H10	111.4
C6—C7—C15	113.16 (13)	C11—C10—H10	111.4
C8—C7—C11	97.80 (12)	O3—C12—H121	120.0
C8—C7—C15	110.62 (15)	O3—C12—H122	120.0
C11—C7—C15	114.07 (14)	C11—C12—H121	120.0
O2—C8—C7	117.07 (15)	C11—C12—H122	120.0
O2—C8—C9	109.54 (14)	H121—C12—H122	109.5
C7—C8—C9	105.64 (15)	C3—C13—H131	109.5
C8—C9—C10	105.72 (13)	C3—C13—H132	109.5
O1—C10—C9	112.64 (14)	C3—C13—H133	109.5
O1—C10—C11	108.15 (12)	H131—C13—H132	109.5
C9—C10—C11	101.46 (15)	H131—C13—H133	109.5
O3—C11—C7	118.26 (15)	H132—C13—H133	109.5
O3—C11—C10	113.99 (12)	C6—C14—H141	109.5
O3—C11—C12	59.75 (11)	C6—C14—H142	109.5
C7—C11—C10	103.95 (13)	C6—C14—H143	109.5
C7—C11—C12	129.49 (14)	H141—C14—H142	109.5
C10—C11—C12	123.35 (16)	H141—C14—H143	109.5
O3—C12—C11	60.11 (11)	H142—C14—H143	109.5
C8—O2—H201	110.7	C7—C15—H151	109.5
O1—C1—H1	108.5	C7—C15—H152	109.5
C2—C1—H1	108.5	C7—C15—H153	109.5
C6—C1—H1	108.5	H151—C15—H152	109.5
C1—C2—H2	117.8	H151—C15—H153	109.5
C3—C2—H2	117.8	H152—C15—H153	109.5
C3—C4—H41	108.5		
			
C1—O1—C10—C9	−48.89 (16)	C14—C6—C7—C11	−179.10 (14)
C1—O1—C10—C11	62.42 (17)	C14—C6—C7—C15	54.7 (2)
C10—O1—C1—C2	−175.48 (11)	C6—C7—C8—O2	40.8 (2)
C10—O1—C1—C6	−51.55 (16)	C6—C7—C8—C9	−81.40 (17)
C12—O3—C11—C7	121.49 (16)	C6—C7—C11—O3	−163.39 (12)
C12—O3—C11—C10	−115.92 (18)	C6—C7—C11—C10	69.08 (15)
O1—C1—C2—C3	105.4 (2)	C6—C7—C11—C12	−90.7 (2)
O1—C1—C6—C5	−71.96 (16)	C8—C7—C11—O3	79.03 (15)
O1—C1—C6—C7	48.78 (17)	C8—C7—C11—C10	−48.50 (15)
O1—C1—C6—C14	169.78 (14)	C8—C7—C11—C12	151.67 (18)
C2—C1—C6—C5	48.01 (18)	C11—C7—C8—O2	152.82 (15)
C2—C1—C6—C7	168.75 (14)	C11—C7—C8—C9	30.61 (16)
C2—C1—C6—C14	−70.25 (19)	C15—C7—C8—O2	−87.74 (19)
C6—C1—C2—C3	−17.7 (2)	C15—C7—C8—C9	150.05 (15)
C1—C2—C3—C4	−3.0 (3)	C15—C7—C11—O3	−37.8 (2)
C1—C2—C3—C13	175.8 (2)	C15—C7—C11—C10	−165.28 (15)
C2—C3—C4—C5	−9.8 (3)	C15—C7—C11—C12	34.9 (2)
C13—C3—C4—C5	171.3 (2)	O2—C8—C9—C10	−129.73 (15)
C3—C4—C5—C6	43.1 (2)	C7—C8—C9—C10	−2.80 (18)
C4—C5—C6—C1	−61.64 (19)	C8—C9—C10—O1	88.78 (16)
C4—C5—C6—C7	179.67 (15)	C8—C9—C10—C11	−26.62 (17)
C4—C5—C6—C14	56.3 (2)	O1—C10—C11—O3	159.65 (14)
C1—C6—C7—C8	47.34 (19)	O1—C10—C11—C7	−70.22 (17)
C1—C6—C7—C11	−59.22 (16)	O1—C10—C11—C12	91.20 (19)
C1—C6—C7—C15	174.58 (15)	C9—C10—C11—O3	−81.67 (17)
C5—C6—C7—C8	164.80 (14)	C9—C10—C11—C7	48.45 (16)
C5—C6—C7—C11	58.24 (16)	C9—C10—C11—C12	−150.13 (16)
C5—C6—C7—C15	−68.0 (2)	C7—C11—C12—O3	−103.3 (2)
C14—C6—C7—C8	−72.54 (18)	C10—C11—C12—O3	100.35 (17)

### Hydrogen-bond geometry (Å, °)

**Table:**

*D*—H···*A*	*D*—H	H···*A*	*D*···*A*	*D*—H···*A*
O2—H201···O1^i^	0.84	2.02	2.839 (2)	165

Symmetry codes: (i) *x*, *y*+1, *z*.
